# Anticancer Activity of γ-Bisabolene in Human Neuroblastoma Cells via Induction of p53-Mediated Mitochondrial Apoptosis

**DOI:** 10.3390/molecules21050601

**Published:** 2016-05-07

**Authors:** Yu-Jen Jou, Chun-Hung Hua, Chen-Sheng Lin, Ching-Ying Wang, Lei Wan, Ying-Ju Lin, Su-Hua Huang, Cheng-Wen Lin

**Affiliations:** 1 Department of Medical Laboratory Science and Biotechnology, China Medical University, No. 91, Hsueh-Shih Road, Taichung 404, Taiwan; alvajou@gmail.com (Y.-J.J.); spirit1126@hotmail.com (C.-Y.W.); 2 Department of Otolaryngology, China Medical University Hospital, Taichung 404, Taiwan; flowererix@yahoo.com.tw; 3 Division of Gastroenterology, Kuang Tien General Hospital, Taichung 433, Taiwan; b8401126@yahoo.com.tw; 4 Department of Medical Genetics and Medical Research, China Medical University Hospital, Taichung 404, Taiwan; lei.joseph@gmail.com (L.W.); yjlin.kath@gmail.com (Y.-J.L.); 5 Department of Biotechnology, College of Health Science, Asia University, Wufeng, Taichung 413, Taiwan

**Keywords:** γ-bisabolene, neuroblastoma, anticancer, apoptosis, p53, CK2α

## Abstract

γ-Bisabolene has demonstrated antiproliferative activities against several human cancer cell lines. This study first discloses the antiproliferative and apoptosis induction activities of γ-bisabolene to human neuroblastoma TE671 cells. A CC_50_ value of γ-bisabolene was 8.2 μM to TE671 cells. Cell cycle analysis with PI staining showed γ-bisabolene elevating the sub-G1 fractions in a time-dependent manner. In addition, annexin V-FITC/PI staining showed γ-bisabolene significantly triggering early (annexin-V positive/PI negative) and late (annexin-V positive/PI positive) apoptosis in dose-dependent manners. γ-Bisabolene induced caspase 3/8/9 activation, intracellular ROS increase, and mitochondrial membrane potential decrease in apoptosis of human neuro-blastoma cells. Moreover, γ-bisabolene increased p53 phosphorylation and up-regulated p53-mediated apoptotic genes Bim and PUMA, as well as decreased the mRNA and protein levels of CK2α. Notably, the results indicated the involvement of CK2α-p53 pathways in mitochondria-mediated apoptosis of human neuroblastoma cells treated with γ-bisabolene. This study elucidated the apoptosis induction pathways of γ-bisabolene-treated neuroblastoma cells, in which could be useful for developing anti-neuroblastoma drugs.

## 1. Introduction

Neuroblastoma is a tumor of neural progenitor cells, which widely metastasizes to skin, bone marrow, liver, and non-contiguous lymph nodes, and then becomes the most common extracranial solid tumor in children [1]. Neuroblastoma is found in a greater than 7% of malignancies and near 15% of oncology deaths in children [2]. The infant patients with metastasis only to skin, bone marrow, and liver have the good prognosis post treatment with the current anticancer drugs. The 5-year event-free survival rate of children with disseminated neuroblastoma (Stage 4) is still less than 40% post-treatment [3,4]. In addition, neuroblastoma has resistance to chemotherapeutic drugs, and even recurrence [2]. Therefore, the development of novel anti-neuroblastoma agents is still recommended to improve the patient outcomes.

Apoptotic inducers have been widely regarded as potential anti-cancer therapeutics and evaluated in pre-clinical or clinical trials [5]. Apoptosis results from initiating Fas receptor-mediated or mitochondria-dependent pathways, subsequently converting DNA fragmentation, cytoskeletal protein degradation, and apoptotic body formation [6]. Anti- and pro-apoptotic molecules included Bcl-2, BclXL, Bax, Bak, BAD, BIM, and BclXS, regulate in mitochondria-dependent apoptotic pathways [6]. Tumor suppressor protein p53 with proapoptotic activity is the key transcription factor for up-regulation of pro-apoptotic genes like PUMA and Noxa.

γ-Bisabolene shows *in vitro* and *in vivo* antiproliferative and apoptotic activities against human oral squamous cell carcinoma [7]. γ-Bisabolene induces the apoptosis of oral squamous cell carcinoma via p53-medaited signaling pathways. This study further investigates the antiproliferative and apoptotic mechanisms of γ-bisabolene against human neuroblastoma.

## 2. Results

### 2.1. Growth Inhibition and Apoptosis Induction of γ-Bisabolene to Human Neuroblastoma

**Figure 1:**
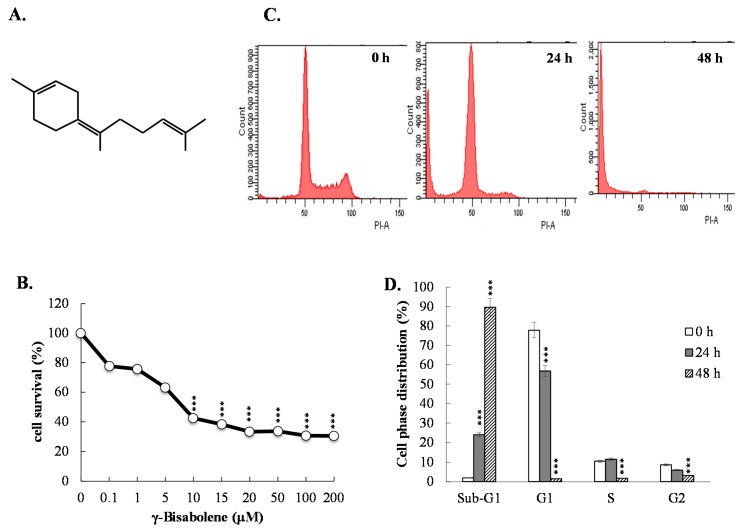
Survival rates and cell cycle analysis of human neuroblastom cells in response to γ-bisabolene. The structure of γ-bisabolene ((*Z*)-1-Methyl-4-(6-methylhept-5-en-2-ylidene) cyclohex-1-ene) was shown (**A**). For antiproliferative assay (**B**), TE671 cells were grown in the indicated concentrations of γ-bisabolene for 48 h. Survival rate was calculated based on MTT assays; For cell cycle analysis (**C**), treated cells with 20 µM γ-bisabolene were fixed by 70% ethanol, incubated with PI solution, then examined using flow cytometry; The percentage of cell distribution was represented (**D**). ***, *p* value < 0.001 compared with untreated cells.

To examine the growth inhibitory ability of γ-bisabolene, the survival rates of human neuroblastoma TE671 cells were examined using MTT assays 2 days post-treatment (Figure 1B). γ-Bisabolene inhibited the growth of TE671 cells in a concentration-dependent manner, exhibiting a CC_50_ value of 8.2 μM (Figure 1B).

Meanwhile, cell cycle analysis of flow cytometry with PI staining showed the increase in the sub-G1 fractions and the decrease in the G1 fractions of γ-bisabolene-treated cells compared to mock controls in a time-dependent manner (Figure 1C,D). The results indicated anti-proliferative activity of γ-bisabolene to human neuroblastoma cells.

### 2.2. Apoptosis of Neuroblastoma Cells Induced by γ-Bisabolene

**Figure 2:**
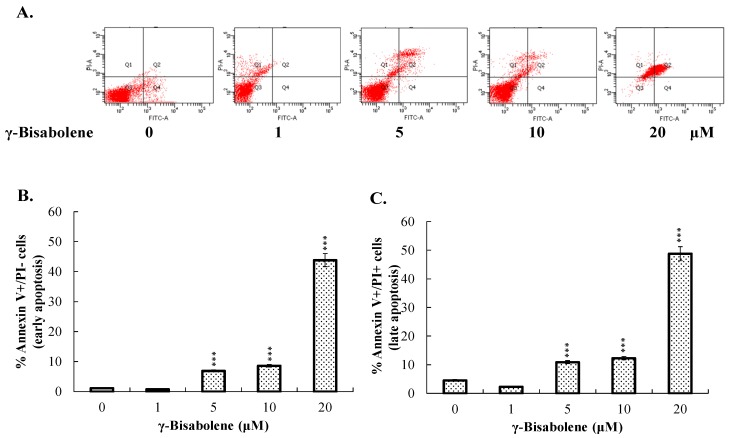
Apoptosis analysis of human neuroblastom cells in responses to γ-bisabolene. Cells were harvested 48 h post treatment, stained by Annexin V-FITC/PI dye, and then analyzed using flow cytometry (**A**); Annexin V positive/PI negative indicated early phase of apoptosis (**B**); Annexin V positive/PI positive presented as late apoptosis (**C**). ***, *p* value < 0.001 compared with untreated cells.

**Figure 3:**
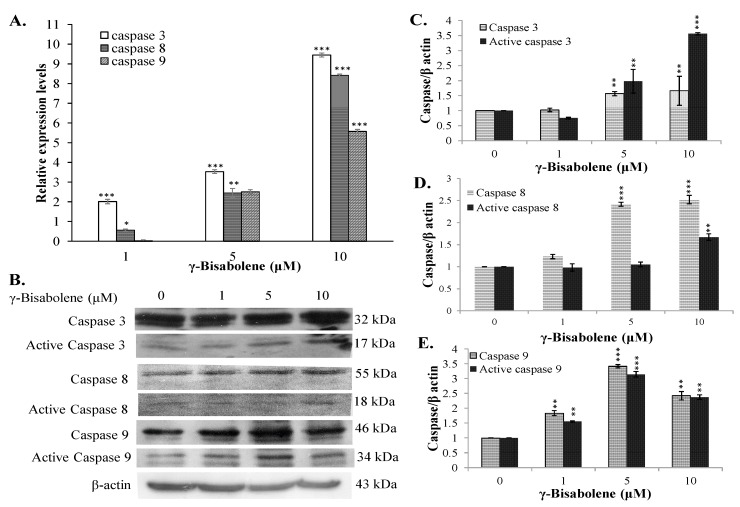
Relative mRNA and protein levels of caspases-3, 8, and 9 in γ-bisabolene-treated cells. Cells were harvested for total RNA extraction and western blotting 48 h post treatment. The relative gene expression was normalized to GAPDH in real-time PCR assays (**A**); Active forms of caspases 3, 8 and 9 in TE671 were characterized using western blotting (**B**); Relative band intensity of indicated caspase or active caspase was normalized by β actin, compared to the mock cells, and quantified using image J based on triplicate replicates of each experiment (**C**–**E**). **, *p* value < 0.01; ***, *p* value < 0.001 compared with untreated cells.

To test whether γ-bisabolene induces apoptosis of human neuroblastoma cells, the fractions of early (annexin-V positive/PI negative) and late (annexin-V positive/PI positive) apoptosis in treated were determined by flow cytometer with annexin V-FITC and PI staining (Figure 2A–C). γ-Bisabolene triggered the significant increase of early and late apoptosis on TE671 cells in dose-dependent manners. To further examine the mRNA and protein levels of caspases 3, 8 and 9 in treated cells, TE671 cells treated were harvested 48 h post treatment for total RNA extraction and the lysate preparation. Quantitative RT-PCR revealed that γ-bisabolene significantly induced the mRNA expression of caspases 3, 8, and 9 in dose-dependent manners (Figure 3A). Caspases 3, 8 and 9 were activated to greater than 5 folds in response to 10 µM γ-bisabolene. Subsequently, western blots indicated the dose-dependent increase in pro- and active forms of caspases 3, 8, and 9 in γ-bisabolene-treated cells 48 h post treatment (Figure 3B–E). Active forms of caspases 3, 8, and 9 exhibited 3.5-, 1.6-, and 2.3-fold increases post-treatment with 10 µM γ-bisabolene, respectively. The results demonstrated γ-bisabolene induces extrinsic and intrinsic apoptosis of human neuroblastoma.

### 2.3. ROS Production Increase and Mitochondrial Membrane Potential Decrease in Treated Cells

**Figure 4:**
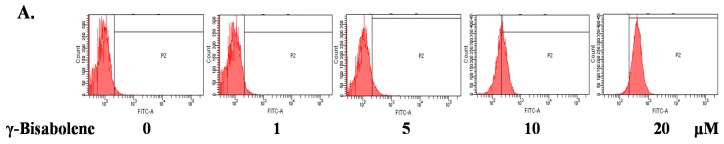

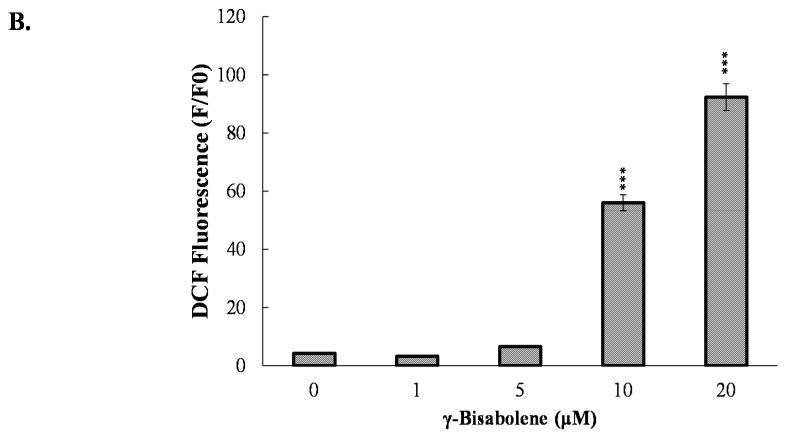
Increase of intracellular ROS production in γ-bisabolene-treated cells. Cells were treated with γ-bisabolene for 48 h, harvested stained using DCF-DA, and then analyzed by flow cytometry with excitation and emission spectra of 495 nm and 529 nm respectively (**A**); Relative fluorescent intensity of DCF was further calculated (**B**). ***, *p* value < 0.001 compared with untreated cells.

**Figure 5:**
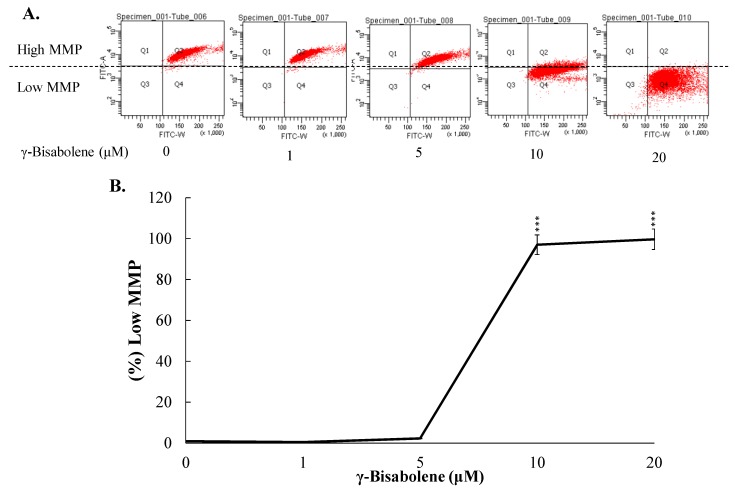
Loss of mitochondrial membrane potential (ΔΨM) in human neuroblastom cells treated with γ-bisabolene. TE671 cells were stained using DiOC6(3), and then measured by flow cytometry (**A**); Relative changes in low MMP of cells treated with γ-bisabolene were shown (**B**). ***, *p* value < 0.001 compared with untreated cells.

To examine the apoptotic pathways of γ-bisabolene-induced apoptosis, the changes in the intracellular reactive oxygen species (ROS) levels and mitochondrial membrane potential (MMP) in γ-bisabolene-treated cancer cells was subsequently surveyed using flow cytometry analysis with DCFH-DA and DiOC6(3) staining, respectively (Figure 4 and Figure 5). γ-Bisabolene treatment induced ROS production in human neuroblastom cells in a dose-dependent manner (Figure 4).

Quantitative analysis of MMP changes using DiOC6(3) staining demonstrated that γ-bisabolene significantly decreased the MMP of human neuroblastom cells (Figure 5A,B); a 97% decrease in MMP was found in TE671 cells treated with 5 μM of γ-bisabolene (Figure 5B). Therefore, the results indicated γ-bisabolene induces ROS production and injures the mitochondria in apoptosis of human neuroblastom cells.

### 2.4. The Increase of p53-Mediated Transcription in γ-Bisabolene-Treated Cells

**Figure 6:**
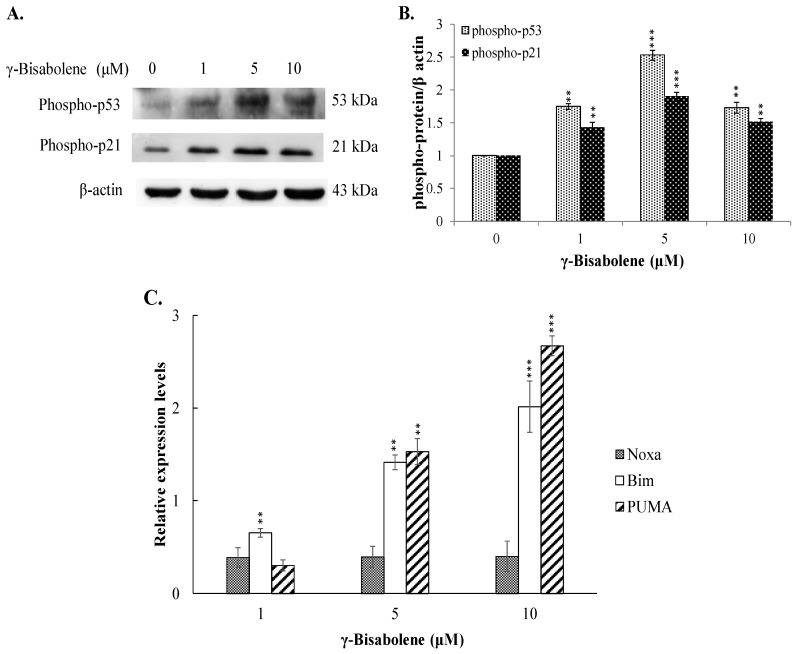
Activation of p53 mediated transcriptional activity by γ-bisabolene. Western blot analysis of lysates from treated and mock cells was performed; the blots were probed with primary and secondary antibodies (**A**); Relative band intensity of phospho-p53 or phosphor-p21 was normalized by β actin, compared to the mock cells, and quantified using imageJ based on triplicate replicates of each experiment (**B**); Relative fold levels in treated and mock cells appear as ratio of indicated mRNA/GAPDH mRNA after performing real time PCR assays (**C**). **, *p* value < 0.01; ***, *p* value < 0.001 compared with untreated cells.

**Figure 7:**
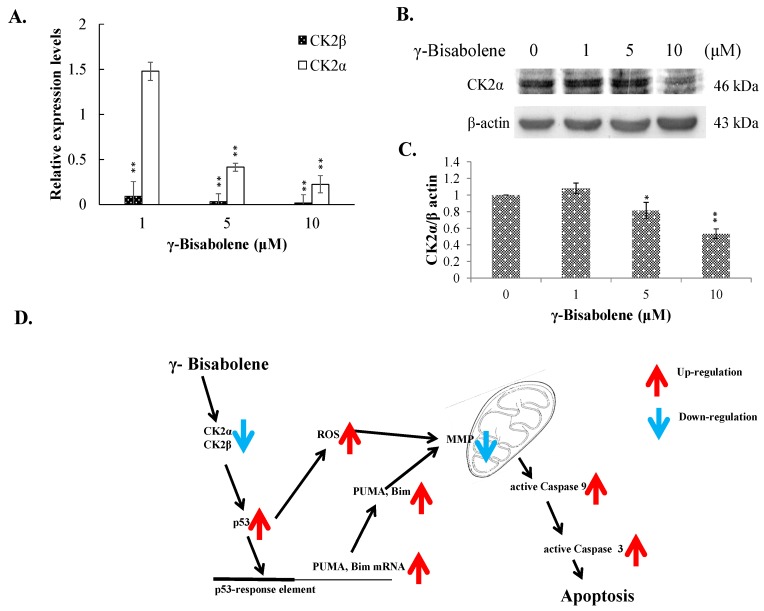
Decrease of CK2α/β expression in γ-bisabolene-treated cells. Cells were treated with γ-bisabolene for 48 h, and then harvested for real time PCR (**A**) and western blot assays (**B**,**C**); Relative mRNA levels of CK2α and CK-2β were normalized by GAPDH, and then compared to un-treated cells, respectively. Relative band intensity of CK2α was normalized by β actin, compared to the mock cells, and quantified using image J based on triplicate replicates of each experiment (**C**); GeneGO Meta-Core pathway analysis of the results predicts p53-mediated apoptosis pathway in treated cells (**D**). *, *p* value < 0.05; **, *p* value < 0.01 compared with untreated cells.

To examine the γ-bisabolene-induced apoptotic pathways of human neuroblastom cells, p53-mediated transcriptional activities for apoptosis induction were subsequently analyzed (Figure 6). Western blotting showed γ-bisabolene elevating the phosphorylation of p53 and p21 in TE671 cells (Figure 6A,B), linked with activation of PUMA and Bim expression, but not Noxa, in dose-dependent manners quantified using real-time RT-PCR (Figure 6C). Since down-regulation of casein kinase 2α (CK2α) was associated with activation of p53 transcriptional activities in apoptosis of γ-bisabolene-treated oral cancer cells [7], the CK2α and CK2β expression in γ-bisabolene-treated neuroblastom cells was further characterized (Figure 7A–C). Real-time RT-PCR and western blotting demonstrated that γ-bisabolene significantly reduced the CK2α and CK2β expression in treated human neuroblastom cells. Therefore, CK2 down-regulation was suggested to be associated with p53-mediated apoptosis of γ-bisabolene-treated neuroblastom cells.

## 3. Discussion

Essential oils containing γ-bisabolene have demonstrated anti-proliferative activities to human oral cancer, prostate cancer, glioblastoma, lung carcinoma, breast carcinoma, and colon adenocarcinoma cell lines [7,8,9,10]. The study first reports the potent antiproliferative and apoptotic activities of γ-bisabolene on human neuroblastom cells (Figure 1, Figure 2 and Figure 3). γ-Bisabolene induced apoptosis of human neuroblastom cells through increasing intracellular ROS, declining MMP, and activating p53-mediated transcriptional activities (Figure 4, Figure 5, Figure 6 and Figure 7). MTT assays indicated γ-bisabolene at high concentrations (15 to 200 µM) was less cytotoxic than low concentrations (0.1 to 10 µM) (Figure 1B), but cell cycle analysis demonstrated sub-G1 fraction as near 90% in treated cells with 20 µM 48 h post treatment (Figure 1C,D). Since the MTT assay relies on mitochondrial dehydrogenase activity, γ-bisabolene at low concentrations (0.1 to 10 µM) was effective at triggering the mitochondria-dependent apoptosis of human neuroblastom cells, as consistent with the findings in Figure 2, Figure 3, Figure 4, Figure 5, Figure 6 and Figure 7.

Tumor suppressor p53 has demonstrated its involvement in the mitochondrial apoptotic pathway, exerting transcriptional activities on regulating the expression of pro-apoptotic genes: e.g., PUMA, Bim, and NOXA [6,11,12]. γ-Bisabolene concentration-dependently increased p53 phosphorylation that linked with up-regulation of p53 transcriptional activities on apoptotic genes PUMA and Bim in human neuroblastom cells (Figure 6). Since PUMA and Bim have been known as the activator for Bax involved in mitochondria-mediated apoptosis [13], γ-Bisabolene-induced p53-mediated PUMA and Bim up-regulation correlated with the MMP decreases in treated neuroblastom cells (Figure 5). In addition, p53 could induce the Fas up-regulation that activates the extrinsic apoptotic pathway [14]. Thus, p53 activation induced by γ-bisabolene might be responsible for increasing the expression and activity of caspase 8 (Figure 3). Results demonstrated γ-bisabolene triggering p53-mediated apoptosis of human neuroblastom cells.

Protein kinase CK2α participates in heart and neural tube development, maintains cell viability, and regulates cell cycle stages [15,16,17,18]. CK2α has been identified to overexpress in human colorectal cancer [19]; gene silence of CK2α small interfering RNA was associated with elevating the expression of p53/p21 and decreasing the expression of C-myc [20]. Therefore, suppression of CK2α was suggested to a novel therapeutic approach for human colorectal cancer. This study demonstrated γ-bisabolene down-regulating the CK2α and CK2β expression in human neuroblastom cells (Figure 7A–C). Thus, down-regulation of CK2 was proposed to as one of promising mechanisms for activating p53-mediated apoptosis of human neuroblastom cells treated with γ-bisabolene (Figure 7D).

## 4. Materials and Methods

### 4.1. Cell Cultures

Human neuroblastoma TE671 cells were cultured in MEM medium (HyClone Laboratories, South Logan, UT, USA) supplemented with 10% fetal bovine serum, 100 U/mL penicillin, 100 μg/mL streptomycin, 2 mM glutamine and 1 mM sodium pyruvate at 37 °C in a humidified atmosphere of 5% CO_2_.

### 4.2. In Vitro Cytotoxicity Test

Cytotoxic activity of γ-bisabolene (4-(1,5-dimethyl-4-hexenylidene)-1-methylcyclohexene) (Figure 1A) was assessed by MTT assay. Cells (5 × 10^4^ cells/mL) plated in 96-well plates overnight and then treated with indicated concentrations (0.1, 1, 5, 10, 15, 20, 50, 100, and 200 μM) for 48 h at 37 °C in humidified atmosphere of 5% CO_2_. Cell were incubated with 10 μL of a MTT solution for 4 h, reacted with 100 μL of stop solution for dissolving formazan crystals, and then measured with micro-ELISA reader by optical density (OD) at 570–630 nm. 50% cytotoxic concentration (CC_50_) of γ-bisabolene on TE671 cells was determined using the ID_50_ software developed by Dr. John Spouge with the survival rate of treated cells to mock cells.

### 4.3. Apoptosis and Cell Cycle Assay by Flow Cytometry

Cells were treated with γ-bisabolene at 10 μM for 24 and 48 h, and harvested for cell cycle analysis by propidium iodide (PI) staining, and apoptosis assays with γ-bisabolene at 1, 5, 10, and 20 μM for 48 h by Annexin V-FITC apoptosis Detection Kit with PI (BioVision, Milpitas, CA, USA). Cell cycle and apoptosis assays were performed as described in our prior report [7].

### 4.4. Mitochondrial Membrane Potential (MMP) Detection Assays

Cells were treated with γ-bisabolene at 1, 5, 10, and 20 μM for 48 h; MMP changes were quantitated using flow cytometry with DiOC6(3) staining (Calbiochem, San Diego, CA, USA). Cells were stained with the DiOC6(3) solution at 37 °C in the dark for 1 h, and then measured using a flow cytometer with an excitation wavelength of 488 nm and an emission wavelength of 530 nm.

### 4.5. Detecting Intracellular Reactive Oxygen Species (ROS) by Flow Cytometry

Cells were treated with γ-bisabolene at 1, 5, 10, and 20 μM for 48 h, harvested, and then stained with 10 μM 2,7-dichlorodihydrofluorescein diacetate (DCFH-DA, Sigma, Saint Louis, MO, USA) at 37 °C for 30 min in darkroom. DCF fluorescence converted from DCFH-DA by ROS was detected by flow cytometry with excitation wavelength of 485 nm and emission wavelength of 530 nm, as described in our prior report [21].

### 4.6. Western Blot

TE671 cells were treated with γ-bisabolene at 1, 5 and 10 μM for 48 h, collected, and lysed in the RIPA lysis buffer (Roche Diagnostics, New York, NY, USA). Lysates in sample buffer were boiled for 8 min, separated by 10% sodium dodecyl sulfate-polyacrylamide gel electrophoresis (SDS-PAGE), and then followed by western blotting assays, as described in our prior report [7,22]. The blots were probed with specific primary antibodies against caspase 3 (Calbiocem), capspase 8, caspase 9 (upstate), phospho-p53(S15), p21 (Millipore, Billerica, MA, USA), phospho-p21(T145) (ABGENT, San Diego, CA, USA), casein kinase 2α (CK2α), and β-actin (Cell Signalling, Danvers, MA, USA), and then reacted with HRP-conjugated anti-mouse or anti-rabbit IgG antibodies (Invitrogen, Carlsbad, CA, USA). Immunoreactive bands were reacted with chemiluminescent western blotting detection reagents, and then pictured by autoradiography.

### 4.7. Real-Time RT-PCR

Total RNA was extracted from mock control and treated cells with γ-bisabolene using the RNA purification kit (Invitrogen), and then converted into cDNA with oligo dT primer and SuperScript III reverse transcriptase (Thermo Fisher, San Jose, CA, USA). Two-step RT-PCR was performed using SYBR Green I, as described in our prior report [23]. Oligonucleotide primer pairs in our study were listed as the following: (1) forward primer 5′-CAGTGGAGGCCGACTTCTTG-3′ and reverse primer 5′-TGGCACAAAGCGACTGGAT-3′ for human caspase 3; (2) forward primer 5′-TGTCCTACTCTACTTTCCCAGGTTTT-3′ and reverse primer 5′-GTGAGCCCACTGCTCAAAGAT-3′ for human caspase 9; (3) forward primer 5′-AGTGGGTATTTCTCTTTTGACACAG-3′ and reverse primer 5′-GTCTCCAATACGCCGCAACT-3′ for human Bim; (4) forward primer 5′-GACGACCTCAACGCACAGTA-3′ and reverse primer 5′-CACCTAATTGGGCTCCATCT-3′ for human PUMA; (5) forward primer 5′-GAGATGCCTGGGAAGAAGG-3′ and reverse primer 5′-TTCTGCCGGAAGTT CAGTTT-3′ for human Noxa; (6) forward primer 5′-GGATGGCCACTGTGAATAACTG-3′ and reverse primer 5′-TCGAGGACATCGCTCTCTCA-3′ for caspase 8; (7) forward primer 5′-GAACGCTTTGTCCACAGTGA-3′ and reverse primer 5′-TATCGCAGCAGTTTGTCCAG-3′ for human protein kinase CK2α; (8) forward primer 5′-CCCATTGGCCTTTCAGACAT-3′ and reverse primer 5′-CCGTGTGATGGTGTCTTGATG-3′ for CK2β; and (9) forward primer 5′-CCACCCATGGCAAATTCC-3′ and reverse primer 5′-TGGGATTTCCATTGATGACAAG-3′ for human GAPDH. Real-time PCR reaction mixture contained 2 μL of cDNA (reverse transcription mixture), 200 nM of each primer pair in SYBR Green I master mix (LightCycler TaqMAn Master, Roche Diagnostics). Relative mRNA levels of indicated genes were gene normalized by housekeeping gene GAPDH.

### 4.8. Statistical Analysis

All data from three independent experiments were used for measuring mean ± standard error (mean ± S.E.) that was compared using Student’s *t*-test. *p* < 0.05 was considered as statistically significant.

## 5. Conclusions

This study demonstrated that γ-bisabolene has potential to inhibit the growth and inducing apoptosis of human neuroblastom cells. γ-Bisabolene reduced the CK2α expression and activated p53-mediated mitochondrial apoptosis pathway in human neuroblastom cell. The results suggested γ-bisabolene as a potential agent of anti-proliferative and apoptosis-inducing activities for treating human neuroblastom cells.

## References

[1] Park J.R., Eggert A., Caron H. (2008). Neuroblastoma: Biology, prognosis, and treatment. Pediatr. Clin. N. Am..

[2] Maris J.M., Hogarty M.D., Bagatell R., Cohn S.L. (2007). Neuroblastoma. Lancet.

[3] De Bernardi B., Nicolas B., Boni L., Indolfi P., Carli M., Cordero D., Montezemolo L., Donfrancesco A., Pession A., Provenzi M. (2003). Disseminated neuroblastoma in children older than one year at diagnosis: Comparable results with three consecutive high-dose protocols adopted by the Italian Co-Operative Group for Neuroblastoma. J. Clin. Oncol..

[4] Cohn S.L., Pearson A.D., London W.B., Monclair T., Ambros P.F., Brodeur G.M., Faldum A., Hero B., Iehara T., Machin D. (2009). The International Neuroblastoma Risk Group (INRG) classification system: An INRG Task Force report. J. Clin. Oncol..

[5] Liu Q., Wang H.G. (2012). Anti-cancer drug discovery and development: Bcl-2 family small molecule inhibitors. Commun. Integr. Biol..

[6] Elmore S. (2007). Apoptosis: A review of programmed cell death. Toxicol. Pathol..

[7] Jou Y.J., Chen C.J., Liu Y.C., Way T.D., Lai C.H., Hua C.H., Wang C.Y., Huang S.H., Kao J.Y., Lin C.W. (2015). Quantitative phosphoproteomic analysis reveals γ-bisabolene inducing p53-mediated apoptosis of human oral squamous cell carcinoma via HDAC2 inhibition and ERK1/2 activation. Proteomics.

[8] Sylvestre M., Pichette A., Longtin A., Nagau F., Legault J. (2006). Essential oil analysis and anticancer activity of leaf essential oil of Croton flavens L. from Guadeloupe. J. Ethnopharmacol..

[9] Bayala B., Bassole I.H., Gnoula C., Nebie R., Yonli A., Morel L., Figueredo G., Nikiema J.B., Lobaccaro J.M., Simpore J. (2014). Chemical composition, antioxidant, anti-inflammatory and anti-proliferative activities of essential oils of plants from burkina faso. PLoS ONE.

[10] Farag M.A., Al-Mahdy D.A. (2013). Comparative study of the chemical composition and biological activities of Magnolia grandiflora and Magnolia virginiana flower essential oils. Nat. Prod. Res..

[11] Mancini F., Moretti F. (2009). Mitochondrial MDM4 (MDMX): An unpredicted role in the p53-mediated intrinsic apoptotic pathway. Cell Cycle.

[12] Fujita T., Ishikawa Y. (2011). Apoptosis in heart failure. The role of the β-adrenergic receptor-mediated signaling pathway and p53-mediated signaling pathway in the apoptosis of cardiomyocytes. Circ. J..

[13] Shukla S., Sharma A., Pandey V.K., Raisuddin S., Kakkar P. (2016). Concurrent acetylation of FoxO1/3a and p53 due to sirtuins inhibition elicit Bim/PUMA mediated mitochondrial dysfunction and apoptosis in berberine-treated HepG2 cells. Toxicol. Appl. Pharmacol..

[14] Haupt S., Berger M., Goldberg Z., Haupt Y. (2003). Apoptosis - the p53 network. J. Cell Sci..

[15] Seldin D.C., Lou D.Y., Toselli P., Landesman-Bollag E., Dominguez I. (2008). Gene targeting of CK2 catalytic subunits. Mol. Cell. Biochem..

[16] Padmanabha R., Chen-Wu J.L., Hanna D.E., Glover C.V. (1990). Isolation, sequencing, and disruption of the yeast CKA2 gene: Casein kinase II is essential for viability in Saccharomyces cerevisiae. Mol. Cell. Biol..

[17] Buchou T., Vernet M., Blond O., Jensen H.H., Pointu H., Olsen B.B., Cochet C., Issinger O.G., Boldyreff B. (2003). Disruption of the regulatory beta subunit of protein kinase CK2 in mice leads to a cell-autonomous defect and early embryonic lethality. Mol. Cell. Biol..

[18] Kulartz M., Hiller E., Kappes F., Pinna L.A., Knippers R. (2004). Protein kinase CK2 phosphorylates the cell cycle regulatory protein Geminin. Biochem. Biophys. Res. Commun..

[19] Lin K.Y., Tai C., Hsu J.C., Li C.F., Fang C.L., Lai H.C., Hseu Y.C., Lin Y.F., Uen Y.H. (2011). Overexpression of nuclear protein kinase CK2 α catalytic subunit (CK2α) as a poor prognosticator in human colorectal cancer. PLoS ONE.

[20] Zou J., Luo H., Zeng Q., Dong Z., Wu D., Liu L. (2011). Protein kinase CK2α is overexpressed in colorectal cancer and modulates cell proliferation and invasion via regulating EMT-related genes. J. Transl. Med..

[21] Yang T.C., Lai C.C., Shiu S.L., Chuang P.H., Tzou B.C., Lin Y.Y., Tsai F.J., Lin C.W. (2010). Japanese encephalitis virus down-regulates thioredoxin and induces ROS-mediated ASK1-ERK/p38 MAPK activation in human promonocyte cells. Microbes Infect..

[22] Yang T.C., Li S.W., Lai C.C., Lu K.Z., Chiu M.T., Hsieh T.H., Wan L., Lin C.W. (2013). Proteomic analysis for Type I interferon antagonism of Japanese encephalitis virus NS5 protein. Proteomics.

[23] Wang C.Y., Huang S.C., Zhang Y., Lai Z.R., Kung S.H., Chang Y.S., Lin C.W. (2012). Antiviral Ability of Kalanchoe gracilis Leaf Extract against Enterovirus 71 and Coxsackievirus A16. Evid. Based Complement. Altern. Med..

